# Global, regional, and national burden and quality of care index (QCI) of lip and oral cavity cancer: a systematic analysis of the Global Burden of Disease Study 1990–2017

**DOI:** 10.1186/s12903-021-01918-0

**Published:** 2021-11-02

**Authors:** Ahmad Sofi-Mahmudi, Masoud Masinaei, Erfan Shamsoddin, Marcos Roberto Tovani-Palone, Mohammad-Hossein Heydari, Shervan Shoaee, Erfan Ghasemi, Sina Azadnajafabad, Shahin Roshani, Negar Rezaei, Mohammad-Mahdi Rashidi, Reyhaneh Kalantar Mehrjardi, Amir Ali Hajebi, Bagher Larijani, Farshad Farzadfar

**Affiliations:** 1 Non-Communicable Diseases Research Center, Endocrinology and Metabolism Population Sciences Institute, Tehran University of Medical Sciences, Tehran, Iran; 2 Cochrane Iran Associate Centre, National Institute for Medical Research Development (NIMAD), Tehran, Iran; 3 Department of Epidemiology and Biostatistics, Tehran University of Medical Sciences, Tehran, Iran; 4 Ribeirão Preto Medical School, University of São Paulo, São Paulo, 14049-900 Brazil; 5 School of Dentistry, Shahid Beheshti University of Medical Sciences, Tehran, Iran; 6 Elderly Health Research Center, Endocrinology and Metabolism Population Sciences Institute, Tehran University of Medical Sciences, Tehran, Iran; 7 Endocrinology and Metabolism Research Center, Endocrinology and Metabolism Clinical Sciences Institute, Tehran University of Medical Sciences, Tehran, Iran

**Keywords:** Lip and oral cavity cancer, Quality of care, Global burden of disease, DALY

## Abstract

**Background:**

To measure the quality of care for lip and oral cavity cancer worldwide using the data from the Global Burden of Disease (GBD) Study 2017.

**Methods:**

After devising four main indices of quality of care for lip and oral cavity cancer using GBD 2017 study’s measures, including prevalence, incidence, years of life lost, years lived with disability, and disability-adjusted life years, we utilised principal component analysis (PCA) to determine a component that bears the most proportion of info among the others. This component of the PCA was considered as the Quality-of-Care Index (QCI) for lip and oral cavity cancer. The QCI score was then reported in both men and women worldwide and different countries based on the socio-demographic index (SDI) and World Bank classifications.

**Results:**

Between 1990 and 2017, care quality continuously increased globally (from 53.7 to 59.6). In 1990, QCI was higher for men (53.5 for men compared with 50.8 for women), and in 2017 QCI increased for both men and women, albeit a slightly higher rise for women (57.2 for men compared with 59.9 for women). During the same period, age-standardised QCI for lip and oral cavity cancer increased in all regions (classified by SDI and World Bank). Globally, the highest QCI scores were observed in the elderly age group, whereas the least were in the adult age group. Five countries with the least amount of QCIs were all African. In contrast, North American countries, West European countries and Australia had the highest indices.

**Conclusion:**

The quality of care for lip and oral cavity cancer showed a rise from 1990 to 2017, a promising outcome that supports patient-oriented and preventive treatment policies previously advised in the literature. However, not all countries enjoyed such an increase in the QCI to the same extent. This alarming finding could imply a necessary need for better access to high-quality treatments for lip and oral cavity cancer, especially in central African countries and Afghanistan. More policies with a preventive approach and paying more heed to the early diagnosis, broad insurance coverage, and effective screening programs are recommended worldwide. More focus should also be given to the adulthood age group as they had the least QCI scores globally.

## Background

With more than 17.5 million new annual cases and more than 8.5 million annual deaths in 2015 worldwide, cancer is considered one of the leading causes of morbidity and mortality in the post-millennial era [1]. Lip and oral cavity cancer is ranked 16th for both incidence and mortality globally according to the International Agency for Research on Cancer data [2]. It represents the most frequent malignancy in the head and neck region and is usually associated with an aggressive approach and a poor prognosis [3, 4]. Approximately 450,000 new lip and oral cavity cancer cases are diagnosed each year, with only 40–50% of patients surviving for the next five years after the diagnosis [3]. Additionally, due to incremental trends of highly associated risk factors for lip and oral cavity cancer, such as smoking and alcohol consumption, possible surges in their incidence have recently been projected [5]. Overall, lip and oral cavity cancer are perceived as a significant component in the global burden of cancers.

On the other hand, lip and oral cavity cancer are mostly associated with favourable prognosis if diagnosed timely [6]. They are commonly neglected by healthcare professionals outside of dentistry and usually remain undiagnosed until the disease’s final stages [7]. Accordingly, preventive interventions aiming to minimise the risk factors and adjust the lifestyle among populations, including decreased consumption of alcohol, tobacco, and areca nut, have previously been suggested to mitigate the burden of the lip and oral cavity cancer [8]. However, considering the predictions about the rise in oral cancer cases in the near future, boosting care quality and addressing its related issues are inevitably indispensable.

Some efforts have previously been exerted to define a framework to measure the quality of care in cancer patients, and many plausible factors have been expounded in the literature; for instance: pain and distress management, patient satisfaction, feasibility, and accessibility [9–13].

Though, these variables are not easily attainable at a worldwide level, hence the rationale for using indirect variables to estimate the quality of care. From a public health perspective, it is necessary to consider factors that can implicitly explain the quality of care in the patients with lip and oral cavity cancer, namely disability-adjusted life years (DALYs), mortality, prevalence, and incidence of the disease [14]. With a global-scale data set on the quality of care for lip and oral cancers, healthcare policymakers and mid-level care centres can bolster their services and hopefully improve these patients’ quality of care in the future. In this study, we used the data from the GBD Study 2017 to estimate the quality of care for lip and oral cavity cancer between 1990 and 2017 using an original method.

## Methods

Data for this study came from the GBD study 2017 that is publicly available on https://gbd2017.healthdata.org/gbd-search. GBD 2017 provides a standardised approach for estimating incidence, prevalence, deaths, years of life lost (YLLs)—due to premature mortality, years lived with disability (YLDs)—also described as years lived in less-than-ideal health, and DALYs by cause, age groups, sex, year, and location. Full details of the GBD study, including inputs, analytical processes, outputs and methods specific to each cause, are explained elsewhere [15].

This study included lip and oral cavity cancer, identified by International Statistical Classification of Diseases and Related Health Problems, 10th revision (ICD-10) classified in two categories: (a) codes of mortality (C00-C08.9, D10.0-D10.5, D11-D11.9) [16]; and (b) codes of prevalence (C00-C07, C08-C08.9, Z85.81-Z85.810) [17]. These disorders are also presented with B.1.1 code in the GBD database.

We defined four indices related to the quality of care, as follows:

1. $${\text{Prevalence to incidence ratio}} = \frac{{\# {\text{Prevalence}}}}{{\# {\text{Incidence}}}}$$
2. $${\text{Mortality to incidence ratio}} = \frac{{\# {\text{Death}}}}{{\# {\text{Incidence}}}}$$
3. $${\text{DALYs to prevalence ratio}} = \frac{{\# {\text{DALYs}}}}{{\# {\text{Prevalence}}}}$$
4. $${\text{YLLs to YLDs ratio}} = \frac{{\# {\text{YLLs}}}}{{\# {\text{YLDs}}}}$$

Among these indices, the trend of each one can easily be determined. For instance, higher values of prevalence to incidence ratio could mean better care and/or better prevention or even be a sign of decreased lifespan among the lip and oral cavity cancer patients. The mortality to incidence ratio shows the effectiveness of the provided care (if any); thus, the lower the value, the better the effectiveness. DALYs to prevalence ratio is high when a high burden of the disease (due to either mortality or morbidity) is present in the country. YLLs to YLDs ratio signifies the mortality of the disease, meaning that higher figures are representative of a worse off status regarding the survival of cancer patients.

We used principal component analysis (PCA) to unify these indices, a multivariable analytical procedure that extracts linear combinations of variables as orthogonal or uncorrelated components [18]. The first component, a linear combination of all the variables, possesses the better part of the variables’ information. This first-ranked component of the PCA was considered the Quality-of-Care Index (QCI). Component scores were calculated on a scale from 0 to 100 in which higher numbers indicate better status [19, 20].

The distribution was assessed using Socio-demographic Index (SDI) [21]—a summary measure that reflects the development status of a region based on the rankings of average incomes per capita, average educational attainment and fertility rates of all areas in the GBD study—and World Bank classifications for global regions and/or countries. To detect the gender inequality in each country, we used gender disparity ratio (GDR), which simply is the male to female ratio of QCI. Five quintiles of GDR were defined as follows: ≤ 0.5, (0.5 to 0.95], (0.95 to 1.05], (1.05 to 1.5], and > 1.5. We considered the 0.95 to 1.05 quintile as the optimum GDR category. To find countries with a very high or very low QCI compared to others, we used a six-sigma test. Six-sigma approach calculates the mean and standard deviation of the index and specifies values out of the range of (μ − 3σ, μ + 3σ) as the outliers. The outliers (countries) could have two meanings: either a very weak performance or a very good one in a country. Weak performances could occur in specific conditions—such as disease outbreaks, or representing regions where the prevalence of a condition is unnaturally high or low. Full description of the analytical methods used for outlier detection in this study can be found elsewhere [22]. From here onwards, except for the absolute values, all the DALYs rates and QCIs reported in this paper are representing an age-standardised figure. Regarding the age disparity patterns, the QCI for each age group was calculated separately on global and SDI scales. We considered ages under 20 years as “childhood and adolescence”, 20–65 as “adulthood”, and above 65 as “the elderly”.

### Validation

Using a mixed-effect regression model, we considered QCI as a dependent variable while the independent variables were as follows: inpatient and outpatient healthcare utilisation, lip and oral cavity cancer death, prevalence, and attributed death to all risk factors [23]. Considering countries as a random effect, the Pearson’s correlation coefficient between the predicted QCI and healthcare access and quality of care index (HAQI)—an index to appraise the accessibility of care—was calculated to be 0.7 [24]. All the analyses were performed using R 3.6.0 (R Core Team, 2019). Detailed information on our mathematical model’s steps and the statistical protocol are available elsewhere [19, 25].

## Results

### Burden of the lip and oral cavity cancer

The global burden of lip and oral cavity cancer increased from 1990 to 2017, although not steadily. Worldwide, in 2017, lip and oral cavity cancer caused more than 5.2 million DALYs (2.4 million more DALYs compared with 1990) with a death toll of 193,696 (96,204 more deaths compared with 1990). Furthermore, 2.4 (95% uncertainty interval (UI) 2.3–2.5) deaths per 100,000 and 64.2 (60.8–67.1) DALYs per 100,000 were observed in 2017. These rates were higher in men; for example, the DALYs rate in 2017 was over twofold in men than women (87.6 compared to 42.2 per 100,000). DALYs rate for men has decreased in the same time period (91.8 compared to 87.6 per 100,000), whereas has increased for women (37.7 compared to 42.2 per 100,000).

### Quality of care index and gender inequity

**Fig. 1:**
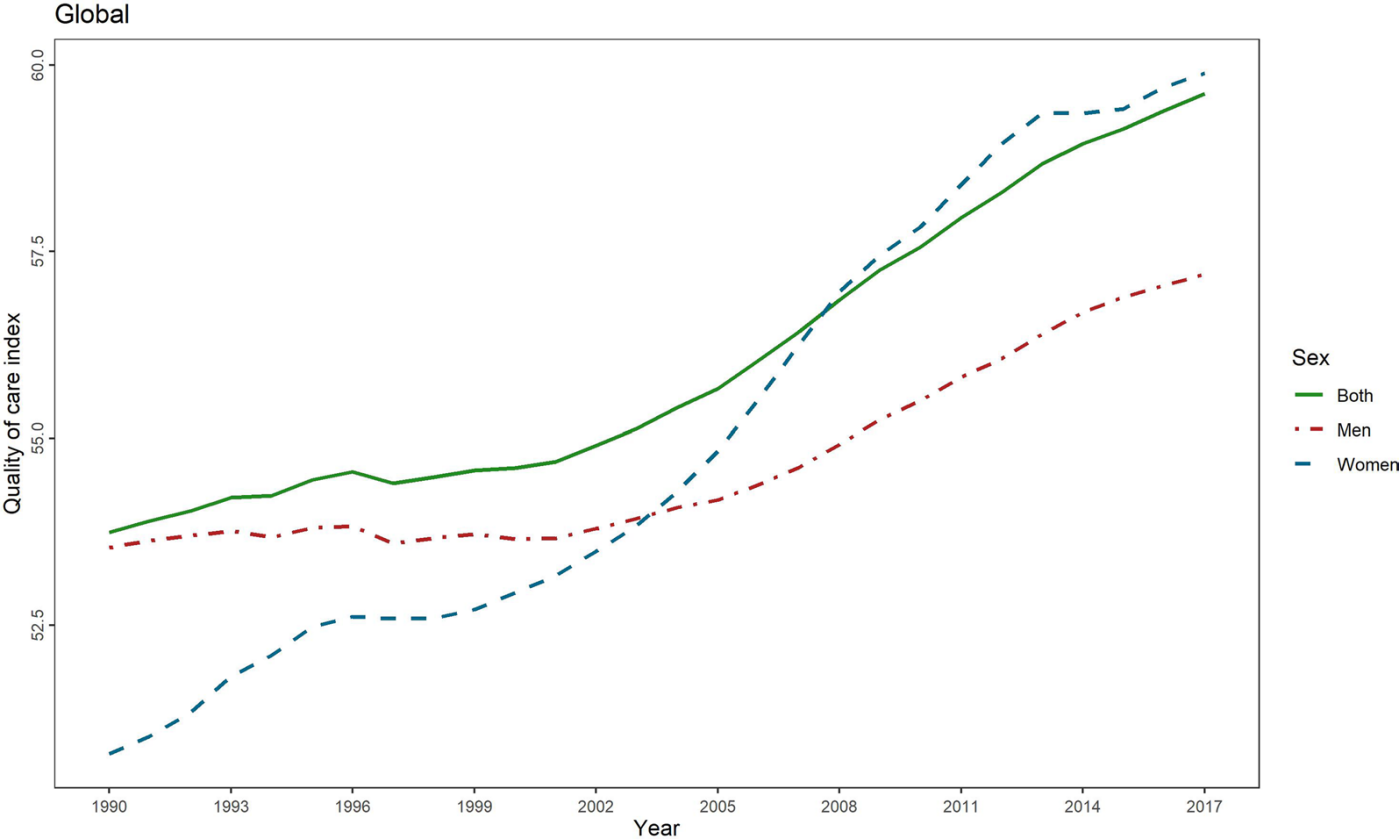
Time pattern of the age-standardised QCI (%) for lip and oral cavity cancer by gender between 1990 and 2017. QCI, Quality of Care Index

**Fig. 2:**
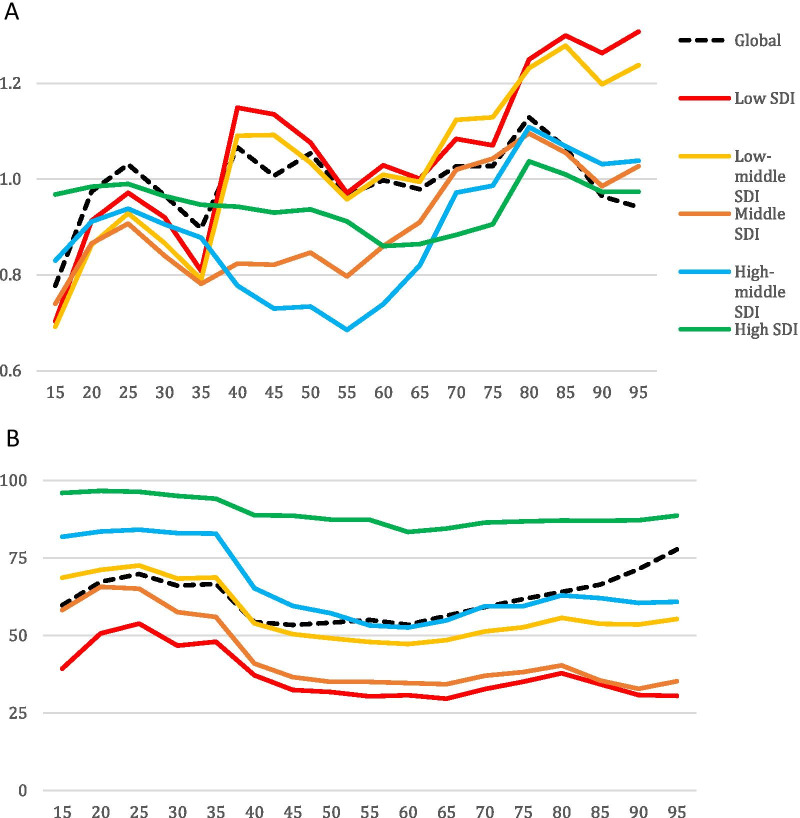
Disparity patterns of the lip and oral cavity cancer in various global and socio-demographic index (SDI) quintile regions in 2017. **A** Vertical axis represents the gender disparity ratio (GDR) in both sexes combined, while the horizontal axis shows the age number. Distinct colours distinguish trends in various SDI quintiles and the global trend. **B** Vertical axis represents the QCI scores (from 0 to 100) in both sexed combined, while the horizontal axis shows the age number. Distinct colours distinguish trends in various SDI quintiles and the global trend

**Fig. 3:**
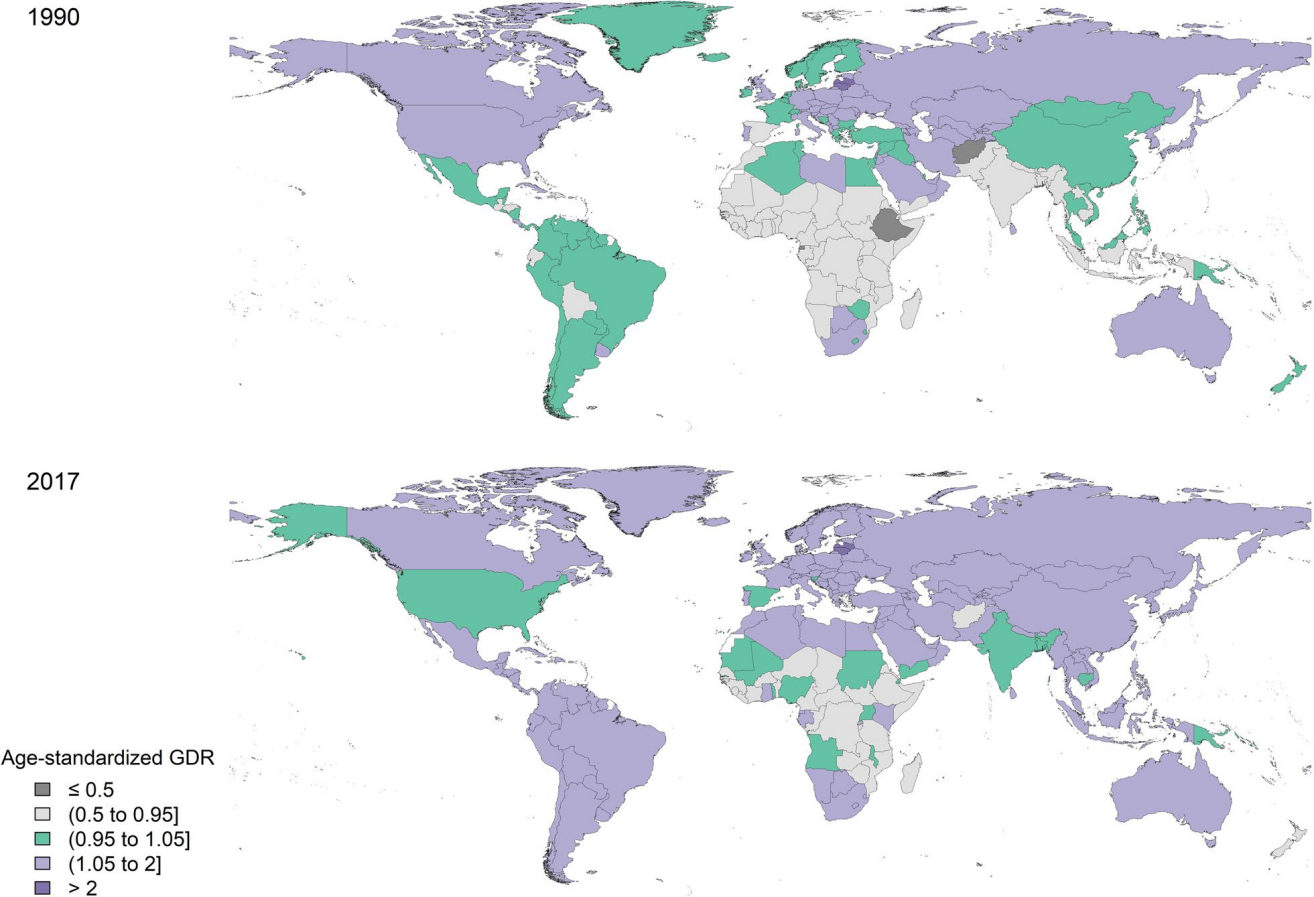
Geographical distribution of GDR for lip and oral cavity cancer. **A** Age-standardised gender disparity ratio in men and women in 1990. **B** Age-standardised gender disparity ratio in men and women in 2017. GDR, Gender disparity ratio

Globally, QCI for lip and oral cavity cancer continuously increased from 1990 to 2017 (from 53.7 to 59.6). In 1990, QCI was higher for men (53.5 for men compared with 50.8 for women), and in 2017 QCI increased for both men and women, albeit a slightly higher rise for women (57.2 for men compared with 59.9 for women) (Fig. 1). On a global scale, gender inequity decreased between men and women from 1990 to 2017, as in 2003, women’s QCIs surpassed that of men and continued to markedly rise till 2017, making women’s superiority even more prominent (Fig. 1). When comparing the GDR among different age groups in various SDI quintiles and worldwide, a fluctuating incremental trend was observed, ranging from 0.068 to 1.31. Investigation of various age groups in 2017 showed the highest QCI scores for the age-group “95 plus” (77.92) and “90–94” (70.86). In the same year, the third-best place concerning the quality of care for lip and oral cavity cancer (67.69) was seen among the “30–34” age group. On the other hand, the least QCI scores in 2017 worldwide belonged to the age group “15–19” (52.36), the adolescents. Generally, high-SDI countries showed higher QCI scores in all age groups compared with the global scores. The other four SDI quintiles, however, entirely or partially fell below the global scores. Figure 2 depicts the QCI and GDR trend among separate age groups in all the SDI quintiles and globally. The global distribution of GDR in men and women in 1990 and 2017 is illustrated in Fig. 3.

### Comparing the countries

Age-standardized QCI for lip and oral cavity cancer increased in all the regions (classified by SDI or World Bank) from 1990 to 2017 (Table 1). Based on World Bank categorisation, high-income countries had the highest QCIs in 2017 (87.8 whilst the lowest DALYs rate was seen in upper-middle-income countries (33.2 per 100,000). On the other hand, low-income countries had the least QCIs in 2017 (33.5), while the highest DALYs rate belonged to lower-middle-income countries. According to SDI classification, high-SDI countries had the highest QCI (88.7) and the least DALYs rate (38.0) in 2017. Low-SDI countries showed the least QCIs in 2017 (34.04). Though, the highest DALYs rate in 2017 belonged to low-middle-SDI countries (125.9) (Table 1).

**Table 1:** Estimates of the burden of disease and QCIs for lip and oral cancers globally and based on the World Bank income classification and SDI quintiles

DALYs rate in 2017 (per 100,000)	DALYs change 1990–2017 (%)	QCI in 2017 (%)	QCI change 1990–2017 (%)
*Global*			
64.23 (60.86, 67.20)	0.58 (− 7.57, 7.27)	59.6	5.9
*World Bank Regions*			
High-income countries			
39.36 (38.15, 40.72)	− 23.88 (− 26.41, − 21.48)	87.85	8.89
Upper-middle-income countries			
33.27 (32.09, 34.41)	− 8.59 (− 12.62, − 4.05)	61.53	15.63
Lower-middle-income countries			
123.83 (114.56, 132.25)	7.04 (− 6.31, 18.55)	45.45	11.95
Low-income countries			
53.09 (49.33, 56.83)	− 5.74 (− 21.77, 10.10)	33.59	5.73
*SDI quintiles*			
High SDI quintile			
38.00 (36.79, 39.33)	− 24.86 (− 27.35, − 22.42)	88.78	8.99
High-middle SDI quintile			
38.63 (37.28, 39.94)	− 13.08 (− 19.43, − 9.06)	61.70	15.11
Middle SDI quintile			
57.09 (53.35, 59.96)	4.31 (− 4.88, 11.75)	53.89	12.65
Low-middle SDI quintile			
125.94 (114.22, 138.75)	12.01 (− 3.64, 28.33)	41.65	8.92
Low SDI quintile			
99.61 (92.48, 106.91)	− 2.56 (− 18.36, 12.56)	34.04	8.02

QCI, Quality of Care Index

Figures in the parentheses show the 95% uncertainty interval (UI)

**Fig. 4:**
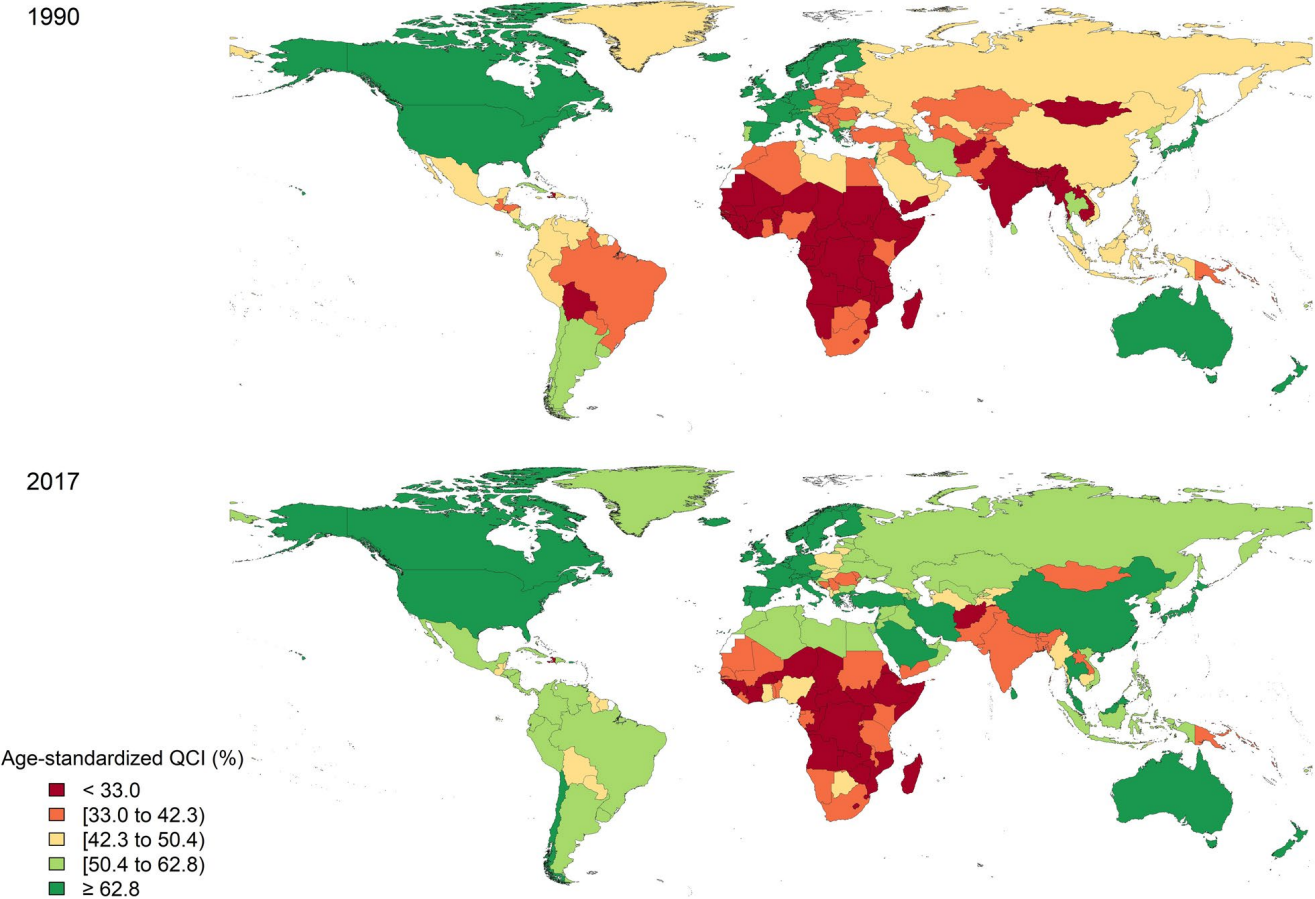
Geographical distribution of QCIs* (%) for lip and oral cavity cancer. **A** Global distribution of age-standardised QCI in men and women in 1990. **B** Global distribution of age-standardised QCI in men and women in 2017. QCI, Quality of Care Index

The top five countries with the least amount of QCIs were all African, while North American countries, West European countries and Australia had the highest indices (Fig. 4). Globally, New Zealand ranked first regarding QCI (100), followed by the United States (99.3), Spain (93.4), United Kingdom (92.4), and Iceland (90.9).

**Fig. 5:**
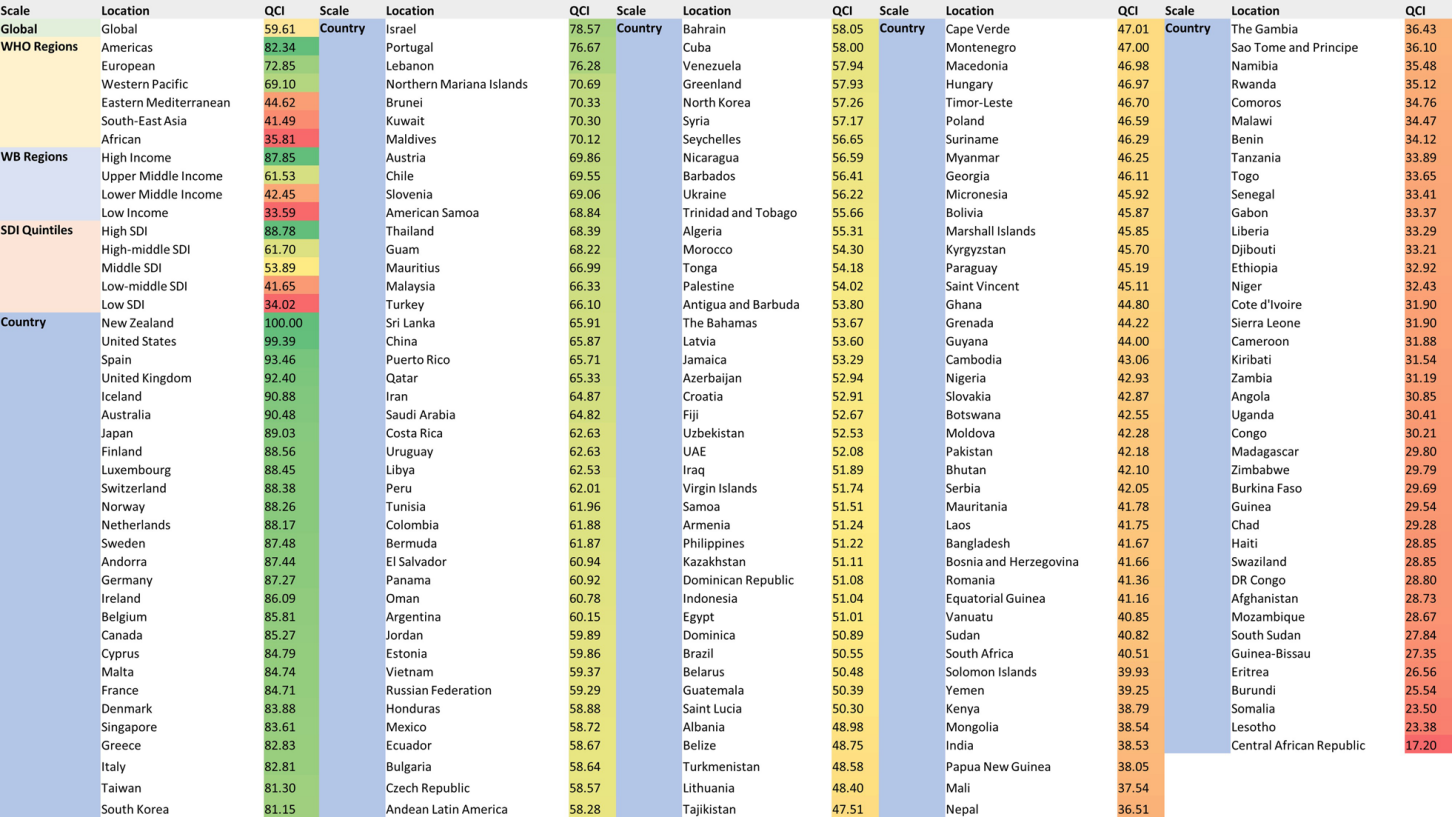
Global regions and countries listed in a descending order based on their QCIs

Shifting to the other side of the QCI spectrum, Central African Republic (17.2), Lesotho (23.3), Somalia (23.5), Burundi (25.5), and Eritrea (26.5) were the countries with the lowest figures. No country’s QCI was at three sigmas lower than the average, whilst the DALYs for Pakistan, Taiwan, and Kiribati were located at three sigmas higher. Figure 5 lists all the countries based on their QCIs.

## Discussion

Using the GBD 2017 data, we devised a multivariable index, the QCI, aiming to represent the quality of care for lip and oral cavity cancer—in men and women—in various regions worldwide from 1990 to 2017. Changes and overall trends of QCI were elucidated as well.

With squamous cell carcinoma making more than 90% of oral cancers, genetic and epigenetic changes, and environmental factors, they are generally deemed the major aetiology of the lip and oral cavity cancer [26–28]. Dietary factors, oral habits (e.g., betel quid chewing), smoking, and alcohol consumption have been listed as other etiologic factors for oral cancers [27, 29]. Therefore, screening programs, diagnostic abilities, and timely provision of care are imperative to decrease the burden of the lip and oral cavity cancer [3, 30–32].

The present study demonstrated that the overall trend of QCI was upwards on a global scale, thereby shifting towards a better quality of care for lip and oral cavity cancer in all the regions according to both SDI and World Bank classifications. When looking at World Bank income groups, the QCIs were all increasing at different paces, an implicit indicator of different access to the most effective treatments and their pre-requisites. Nano-technology-based drug delivery, application of magnetised paclitaxel anti-cancer agents, photodynamic therapy, and chronotherapy are all instances of advanced therapeutic options for lip and oral cavity cancer patients [33–36]. Making these costly combinative treatments available to the public necessitates a wide insurance coverage and great resource allocation, though mainly considered in vain without effective preventive measures [3, 35, 37, 38]. DALYs trends in the corresponding areas, however, were all decreasing unless in lower-middle-income countries. These countries, mostly placed in East Asia and Central Africa, have experienced a rise in DALYs simultaneous to an increase in QCIs. This can be explained as numerous types of smokeless tobacco, and areca nut practices (concerning lip and oral cavity cancer) are quite common among Asian and African residents [39, 40]. Toombak dipping in Sudan is a clear instance of such high-risk behaviours among the public [41].

Turning to SDI classifications, the trend of QCIs was increasing in all the quintiles, again, at different paces. The DALYs rates for low-, high-middle-, and high-SDI quintiles showed a negative change while a positive one was noted in low-middle- and middle-SDI quintiles. The association of socioeconomic status and risk of oral cancer is already illuminated [42]. Accordingly, the decrease in QCIs in countries with lower SDIs is a harbinger of an association between socioeconomic status and the quality of care for lip and oral cavity cancer. The results of QCI analyses were in line with suggestions from a previous study of the burden of oral cancer wherein a decrease in the mortality rate and an increase in the cure rate of oral cancer was proposed globally [39]. These findings are in line with previous studies assessing the QCI for brain and other central nervous system cancers [43], hematologic malignancies [20], thyroid cancer [44], and pancreatic cancer [45], all of which demonstrated higher QCI scores in countries with higher socio-economic status. Since QCI indirectly considers access to care, the positive trends in the quality of care could be considered a promising outcome substantiating patient-oriented policies that have previously been expressed by cancer patients [46].

New Zealand, the United States, Spain, United Kingdom, and Iceland were the leading countries regarding the quality of care for lip and oral cavity cancer patients. All these countries are signposted as high-income economies by the World Bank and high-SDI countries and have established clinical management guidelines for lip and oral cavity cancer. The American Society of Clinical Oncology, for instance, publishes and updates a series of clinical guidelines for the management of head and neck cancers [47]. Applying national screening programmes for lip and oral cavity cancer are still disputed due to their uncommon disposition and lack of cost-efficiency in performing the tests [48, 49]. Nevertheless, dental practitioners and related care providers are emphatically encouraged to screen for any suspicious lesions while examining the oral cavity [48, 50]. The effectiveness of screening programmes is proved to enhance when applied in an opportunistic, high-risk targeting approach [51]. Accordingly, national and regional registration programs are crucial for comprehensive screening, accurate disease monitoring, enhancing the quality of care, and effective policy-making [52–55]. The DALYs’ trend was downwards for all the top-five countries concerning QCI, except for the United Kingdom, which experienced a slight increase in DALYs of the lip and oral cavity cancer between 1990 and 2017. This finding is further substantiated by the increasing trend of overall-5-year survival rate of people with oral or oropharyngeal cancer in many affluent countries. In the US, for instance, this figure has been increasing from 33.3% in the period of 1992–1996 to 65% during 2016–2020 [56, 57]. This is another proof that higher-quality treatment can lower the mortality rate of oral cancers while increasing the quality of life (lower DALYs and YLDs).

Shifting to the other side of the QCI spectrum, the Central African Republic, Lesotho, Somalia, Burundi, and Eritrea were the countries with the lowest figures. All these countries had QCI scores lower than half of the global QCI score in 2017 (= 59.6), meaning that the quality of provided care for lip and oral cavity cancer patients was less than half of the average global. Between 1990 and 2017. Knowing the elevated risk of lip and oral cavity cancer in African countries, mainly due to chewing habits and paucity of oral healthcare workers, it is even of more salience to improve the public access to oral examinations and care provision, whereby the early diagnosis of oral cancers is assured [5, 39, 58–60]. This will most likely lead to an increase in the cure rate and QCI in the corresponding countries [39]. Observing the wide chasm in QCI figures among different regions and countries reiterates the utter importance of equity in healthcare and, in this case, the lip and oral cavity cancer. Afghanistan, a low-income country in South Asia, was the only non-African country placed in the last ten countries concerning QCI figures in 2017. Considering its similar economic situation to the African regions (World Bank classification) and the resultant lack of access to timely treatment for cancer patients, the same rational seems appliable to Afghanistan’s situation. The public in Afghanistan is reported to practice high-risk dietary habits as well; another resemblance to the aetiology of the lip and oral cavity cancer in African countries [61].

Concerning the quality of care and access to oral cancer treatments, our findings show that females had a better status than males globally. This situation can be due to women generally having a more preventive approach for care-seeking (individual aspect) [62], or effective gender-sensitive policy plans towards higher equity [63]. High-SDI countries showed the best performance regarding the age disparities of quality of care for lip and oral cavity cancer with the least amount of QCI variation among different age groups. The other four SDI quintiles depicted more pronounced differences with the best QCIs belonging to those younger than 35, including the childhood and adolescence and the adulthood age groups. Granted, a steady increase was observed in QCI scores in the adult age group; it should be noticed that QCI scores are not optimally reliable in the elderly age group and especially in those older than 80 years [20]. As QCI considers the incidence as a variable, this finding can be expounded by the lower incidence of oral cancer in younger generations [64]. Better access to care [65], exposure to unconventional etiologic factors [66], and higher odds of lack of insurance in younger ages[65] can be mentioned as additional factors. Those between 40 and 60 experience the worst quality of care for oral cancer. This is in line with the higher incidence, prevalence, and mortality rate in this age group for lip and oral cavity cancer [67]. Patients tend to shift toward more palliative treatment with advancing age. This can contribute to the higher mortality rates (increased YLL) and lower QCI scores [67]. Vast screening programs and cancer registry setups could be essential to tackle the issue of early diagnosis in lip and oral cavity cancer cases, ultimately leading to better prognoses and higher quality care [68].

Our findings can assist with the process of health policy decision making and serve as a first-hand guide to compass the future healthcare provision strategies targeting oral cancer in distinct areas.

All in all, tactfully directed policies that address the early prognosis, better resource allocation, higher access, and broader coverage of treatments for the public are imperative to enhance the quality of care in lip and oral cavity cancer patients.

We tried to applicate the most comprehensive measures to capture different aspects of oral healthcare in the context of the GBD database. While doing so, the main factors to consider were DALY, mortality, prevalence, and incidence of the disease. However, other factors influencing the quality of care could not be considered—such as patient satisfaction, staff responsiveness, treatment reliability and validity assessment, etc.—due to the limitations of GBD data. Additionally, the accessibility of healthcare was not considered directly in our index; instead, an indirect correlation was applied to assure the validity of our index in appraising the access. Our results are better to be interpreted cautiously as they are merely estimations and do not represent absolute figures.

## Conclusion

The general trend of quality of care for lip and oral cavity cancer has increased in all the regions globally. However, notable differences have been faced among different countries in terms of quality of care, a harsh harbinger of healthcare inequity. To improve QCIs in the long run, broader insurance coverages and better access to oral healthcare for lip and oral cavity cancer patients are required in countries with lower QCIs, namely, Central Africa, and Afghanistan. More preventive policies, especially in the adult age group, are essential to enhance the quality of care on a global scale.

## Data Availability

The datasets generated during the current study are available upon reasonable request from corresponding author.
